# CCN1 Secretion Induced by Cigarette Smoking Extracts Augments IL-8 Release from Bronchial Epithelial Cells

**DOI:** 10.1371/journal.pone.0068199

**Published:** 2013-07-09

**Authors:** Hyung-Geun Moon, Yijie Zheng, Chang Hyeok An, Yoon-Keun Kim, Yang Jin

**Affiliations:** 1 Division of Pulmonary and Critical Care, Department of Medicine, Brigham and Women’s Hospital, Harvard Medical School, Boston, Massachusetts, United States of America; 2 Department of Life Science and Division of Molecular and Life Sciences, Pohang University of Science and Technology (POSTECH), Pohang, Republic of Korea; Chang Gung University, Taiwan

## Abstract

Inflammation involves in many cigarette smoke (CS) related diseases including the chronic obstructive pulmonary disease (COPD). Lung epithelial cell released IL-8 plays a crucial role in CS induced lung inflammation. CS and cigarette smoke extracts (CSE) both induce IL-8 secretion and subsequently, IL-8 recruits inflammatory cells into the lung parenchyma. However, the molecular and cellular mechanisms by which CSE triggers IL-8 release remain not completely understood. In this study, we identified a novel extracellular matrix (ECM) molecule, CCN1, which mediated CSE induced IL-8 secretion by lung epithelial cells. We first found that CS and CSE up-regulated CCN1 expression and secretion in lung epithelial cells in vivo and in vitro. CSE up-regulated CCN1 via induction of reactive oxygen spices (ROS) and endoplasmic reticulum (ER) stress. p38 MAPK and JNK activation were also found to mediate the signal pathways in CSE induced CCN1. CCN1 was secreted into ECM via Golgi and membrane channel receptor aquaporin4. After CSE exposure, elevated ECM CCN1 functioned via an autocrine or paracrine manner. Importantly, CCN1 activated Wnt pathway receptor LRP6, subsequently stimulated Wnt pathway component Dvl2 and triggered beta-catenin translocation from cell membrane to cytosol and nucleus. Treatment of Wnt pathway inhibitor suppressed CCN1 induced IL-8 secretion from lung epithelial cells. Taken together, CSE increased CCN1 expression and secretion in lung epithelial cells via induction of ROS and ER stress. Increased ECM CCN1 resulted in augmented IL-8 release through the activation of Wnt pathway.

## Introduction

Cigarette smoke (CS) is well known for its association with many respiratory diseases including asthma, emphysema and lung cancer [1], [2]. Exposure to CS leads to the activation of an inflammatory cascade in the upper and lower airway epithelium. In bronchoalveolar lavage fluids (BALF) from smokers, elevated neutrophils, eosinophils and macrophages are found [3], [4]. The accumulation of these inflammatory cells is thought to occur after the release of chemotactic factors from lung epithelial cells in response to CS [5]. Infiltration of inflammatory cells is associated the consequent lung injury and remodeling involved in chronic obstructive pulmonary diseases (COPD) [5]. Therefore, it is important to understand the pathogenesis underlying the CS induced lung inflammation. Airway epithelial cells are known to secrete a variety of pro-inflammatory cytokines and chemokines [6]. For instance, high concentrations of IL-8 have been detected from the induced sputum or BALF obtained from patients with a variety of respiratory conditions, including many CS related diseases, such as chronic obstructive pulmonary diseases (COPD) and chronic airway disease [7], [8].

IL-8 (also named as neutrophil chemotactic factor) belongs to the C-X-C chemokine family and plays a critical role in CS induced respiratory disease [9]. It is not only a neutrophil chemotactic factor, but also a potent activator for T-lymphocytes, eosinophils, basophils and monocytes [10]. CS and cigarette smoke extracts (CSE) both induce the release of IL-8, demonstrated in cultured bronchial epithelial cells in vitro and in BALF from smokers in vivo [9]. It is well accepted by many pulmonologists that CS triggers an inflammatory response by promoting bronchial epithelial cells to release IL-8 [9]. Despite that CS/CSE induced IL-8 from lung epithelial cells has been first demonstrated more than a decade ago, the precise molecular mechanisms and pathways involved in this event remain unclear. Better knowledge on how CS/CSE triggers IL-8 release potentially provides novel therapeutic targets for CS associated lung inflammation.

CCN1, also named Cyr61, belongs to the CCN protein family (**C**yr61, **C**TGF and **N**ov) [11]. CCN1 is a cysteine-rich, 38 kD secreted protein which is expressed in a broad range of cells including lung epithelial cells [12], [13]. As an early stress response gene product and an ECM protein, it plays critical functions in tissue remodeling and repair, including the regulation of apoptosis, differentiation, migration and proliferation [14]. Secreted CCN1 functions in a paracrine and/or autocrine manner (14). It interacts with the integrin family, tyrosine kinase receptor type 1 (TRKA), Wnt and Notch family receptor, to activate the intracellular signaling pathway [11]. A marked induction of CCN1 is detected in gene microarray studies using human tissue from patients with cigarette smoke induced COPD/emphysema [15]. However, the precise biological function of CCN1 in the setting of cigarette smoke exposure and its role in the smoke associated lung inflammation remain unexplored.

Our current study identified that CCN1 plays a crucial role in the process of CSE induced IL-8 release from airway epithelial cells. This finding potentially provides a novel target for therapy and biomarker development on CSE associated lung inflammation.

## Materials and Methods

### Human Lung Tissue

All human lung tissues, as previously reported, were obtained from Lung Tissue Research Consortium. The severity of COPD was classified following the guidelines of the Global Initiative for Obstructive Lung Disease (GOLD) [16].

### Animal

Male 6–8-week old C57BL/6 mice were purchased from Jackson Laboratory (Bar Harbor, ME) and housed under pathogen-free conditions at the animal facility at the Brigham and Women’s Hospital (BWH). All animal experiment protocols were approved by the Harvard Standing Committee for Animal Welfare.

#### In vivo CS exposure

Mice were exposed to CS (100 cigarettes/day for 5 days/wk) for a total of 3 months using a total body CS exposure chamber as described [17] [18]. Total body CS exposure was performed in a stainless-steel chamber (71 cm × 61 cm × 61 cm) using a smoking machine (model TE-10; Teague Enterprises). The smoking machine puffs each 3R4F cigarette for 2 s, for a total of nine puffs before ejection, at a flow rate of 1.05 l/min, providing a standard puff of 35 cm^3^. The smoke machine was adjusted to deliver 10 cigarettes at one time. The chamber atmosphere was periodically measured for total particulate matter and concentrations ranged from 100 to 120 mg/m^3^ (16).

#### Cell culture and treatment

Human bronchial epithelial cell line Beas2B cells were purchased from American Type Tissue Culture Collection (Manassas, VA). Beas2B cells were cultured in 100-mm dishes, containing DMEM with 10%FBS (Life sciences, Grand Island, NY), 2 nM_ L_-glutamine, 100 µg/ml penicillin and 100 U/ml streptomycin, in humidified incubator under 5% CO_2_ at 37°C. Cells were exposed to 10% CSE in all the experiments. For inhibitor treatment, inhibitors were pre-treated 1–3 h followed by CSE exposure.

#### Isolation of Type II lung epithelial cells

The primary alveolar type II (ATII) cells of mouse lung were obtained as previously described [19]. In brief, mice were anaesthetized and the lungs were perfused with cold PBS. Dispase solution (2 ml; Gibco, Grand Island, NY) and 1% low-melting agarose (1 ml; Invitrogen, Grand Island, NY) were infused into tracheal catheter. The lungs were removed and transferred to 2 ml of dispase and incubated on the shaker for 45 min at RT. The lung tissue was teased and filtered through 100 µm and 20 µm strainers, and the pellet was centrifuged. After suspending the cell pellets, the cells were incubated on anti-CD 16/32 plus anti-CD45 pre-coated plates for 1 h at 37°C. The unbounded cells were collected and moved to non-coated plates for 4 h at 37°C, and then the suspending cells were moved to fibronectin (Sigma-aldrich)-coated plates. Three days later, CSE was treated for 24 h.

#### Preparation of cigarette smoke extraction (CSE)

CSE derived from Kentucky Reference 3R4F research blend cigarettes (University of Kentucky) were prepared as described [17]. In brief, CSE was prepared by bubbling smoke from 1 cigarette into 10 ml serum-free DMEM medium and CSE was sterile-filtered through a 0.2 µm filter (VWR International, Radnor, PA).

#### Chemicals and recombinant protein

NAC, ascorbic acid, GSH, brefeldin A, tetraethylammonium chloride (TEA), α18-glycyrrhetinic acid (GA), daidzein, 2-bromopalmitate (2-BP), TGN-020, thapsigargin, 4-Phenylbutyric acid (4-PBA), bay 11-7082 were purchased from Sigma-aldrich (St. Louis, MO), tauroursodeoxycholic acid (TUDCA) from EMD Millipore (Merck KGaA, Darmstadt, Germany), salubrinal from Santa Cruz Biotechnology, JNK inhibitor, SB 203580, γ-secretase inhibitor from Calbiochem (Merck KGaA, Darmstadt, Germany), GSK PERK inhibitor from Toronto Research Chemicals (Toronto, Ontario, Canada). Recombinant human & anti-human CCN1 proteins were purchased from R&D systems (Minneapolis, MN).

#### ELISA

Human CCN1 duoset ELISA was purchased from R&D systems (Minneapolis, MN) and human IL-8 ELISA from Thermo (Rockford, IL), mouse CCN1 ELISA kit from MyBioSource (San Diego, CA) and followed manufacturer’s instruction.

#### Western blot

Cells were harvested after twice cold PBS washing and then suspended in RIPPA buffer with protease inhibitors (Roche, Indianapolis, IN). Total protein samples were resolved by 4–12% NuPAGE gel (Invitrogen, Carlsbad, CA ) and transferred to PVDF membranes (Bio-Rad, Hercules, CA). Membranes were blocked in 5% nonfat milk in PBST for 1 hour at room temperature and then blocked with primary antibodies at 4°C overnight. CCN1 and β-actin antibody were purchased from Santa Cruz (Santa Cruz, CA) and JNK, p-38 MAPK, pJNK, p-p38 MAPK, Ire-1α, p-eIF2α, pLRP6, LPR6, Dvl2, Dvl3, β-catenin, axin, PDI, ERp44, ERp94 and BiP/GRP78 from Cell Signaling (Danvers, MA). Membranes were washed and incubated with appropriate secondary antibodies (Santa Cruz, CA). Detection was performed using the SuperSignal West Pico and Femto system (Pierce, IL) and exposed to Molecular Imager® chemi Doc™ XRS+ (Bio-Rad, Hercules, CA). Normalization and relative quantification were performed with Image Lab software ((Bio-Rad, Hercules, CA).

#### Small interfering RNA (siRNA) transfection

Human Ire-1α siRNA was purchased from Ambion (Life Technologies, Grand Island, NY), human JNK siRNA and p38 MAPK siRNA from Cell Signaling (Danvers, MA) and INTERFERin® from Polyplus (Illkirch, FRANCE). Transfection procedure was performed per INTERFERin® manufacturer’s instruction.

#### Immunohistochemistry (IHC) & Immunofluorescence (IF)

Human lung tissues were fixed with 4% paraformaldehyde and analyzed by immunohistochemical staining for anti-CCN1 (Santa Cruz Biotechnology, Santa Cruz, CA). Briefly, fixed, paraffin-embedded lung tissue sections were deparaffinized with xylene, rehydrated gradually with graded alcohol solutions, and then washed with deionized water. Sections were retrieved by Target Retrieval Solution (Dako). After incubation with 0.3% H2O2 in methanol for 30 min, sections were blocked with blocking serum from Vectastain Elite ABC Kit (Vector Laboratories) for 1 h. Next, the slides were incubated with anti-CCN1 antibody (Santa Cruz Biotechnology, Santa Cruz, CA) at 4°C overnight, followed by incubating with biotinylated universal antibodies for 30 min. Sections were then incubated with Vectastain Elite ABC Reagent for 30 min at room temperature. DAB substrate solution of Peroxidase Substrate Kit (Vector Laboratories) was applied to the sections and counterstained with Hematoxylin QS (Vector Laboratories). After washing with deionized water, sections were dehydrated gradually with graded alcohol solutions and immersed in xylene. Slide sections were mounted with Permount Mounting Medium (Fisher Scientific) and covered with cover glass. For IF staining, formalin-fixed, paraffin-embedded lung tissue sections were subject to IF staining with anti-CCN1 and β-catenin as described previously [17]. Samples were viewed using an Olympus FluoView FV10i confocal laser scanning microscope.

#### ROS detection after CSE treatment

Beas2B cells were stimulated with 10% CSE. After 6 h, cells were detached with trypsin-EDTA. Next, cells were washed with fresh PBS two times and ROS was detected using Image-iT ™ LIVE Green Reactive Oxygen Species Detection Kit (Invitrogen, Carlsbad, CA). ROS level was analyzed using BD FACS Canto II and FlowJo software (Ashland, OR).

### Statistical Analysis (Anova)

The means of fold change in all figures were compared using two-way analysis of variance to test the differences among independent samples. With p<0.05, the difference was considered statistically significant. Error bars indicate the standard deviation.

## Results

### Cigarette Smoke and Cigarette Smoke Extracts (CSE) Induced CCN1 Expression in Lung Epithelial Cells

**Figure 1:**
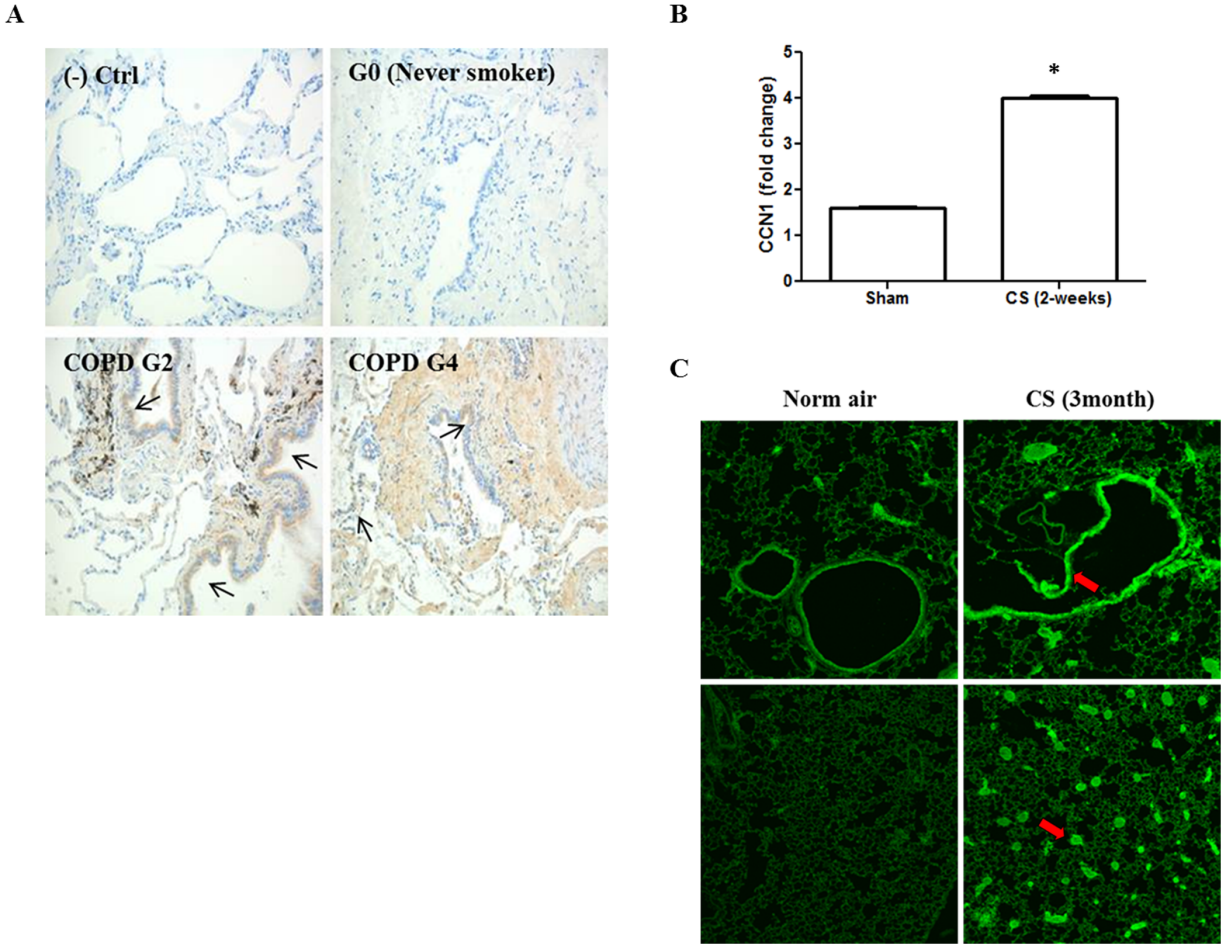
CCN1 expression and secretion after cigarette smoking (CS) and cigarette smoking extracts (CSE). (A) IHC staining for CCN1 expression in human lung tissue obtained from non-smoker (right upper panel), patients in category GOLD 2 (left lower panel) and GOLD 4 (right lower panel) Brown: positive CCN1 (black arrow). (B) CCN1 mRNA transcription after CS in mouse lung tissue. Mice were exposed to CS. After 2 weeks, RNA was isolated from lung tissue and real time PCR was performed. CCN1 mRNA transcription was normalized by β-actin. (C) Mouse lung tissue stained for CCN1 using Immunofluorescence (IF) in the presence and absence of cigarette smoking (3 months), Red arrow: positive CCN1. *p<.05 compared to sham. All figures above represented three independent experiments with similar results.

Initially, we found that CCN1 was highly up-regulated in lung sections obtained from COPD patients (Fig. 1A), comparing with those from the never-smokers (Gold 0). We next exposed C57/Bl6 mice to CS. After two weeks, the RNA from mice lung tissue was isolated. Real time PCR showed an elevated CCN1 mRNA transcription in mice exposed to CS (Fig. 1B). This observation was confirmed using IHC staining on mice lung tissue (Fig. 1C). Increased CCN1 expression was found particularly in airway epithelial cells (red arrow). Lung homogenized tissue from CS exposed mice also indicated higher CCN1 expression (Fig. S1).

### CCN1 Secretion was Augmented by CSE Induced Reactive Oxygen Spices (ROS) via ER-Golgi and Aquaporin4 Pathway

**Figure 2:**
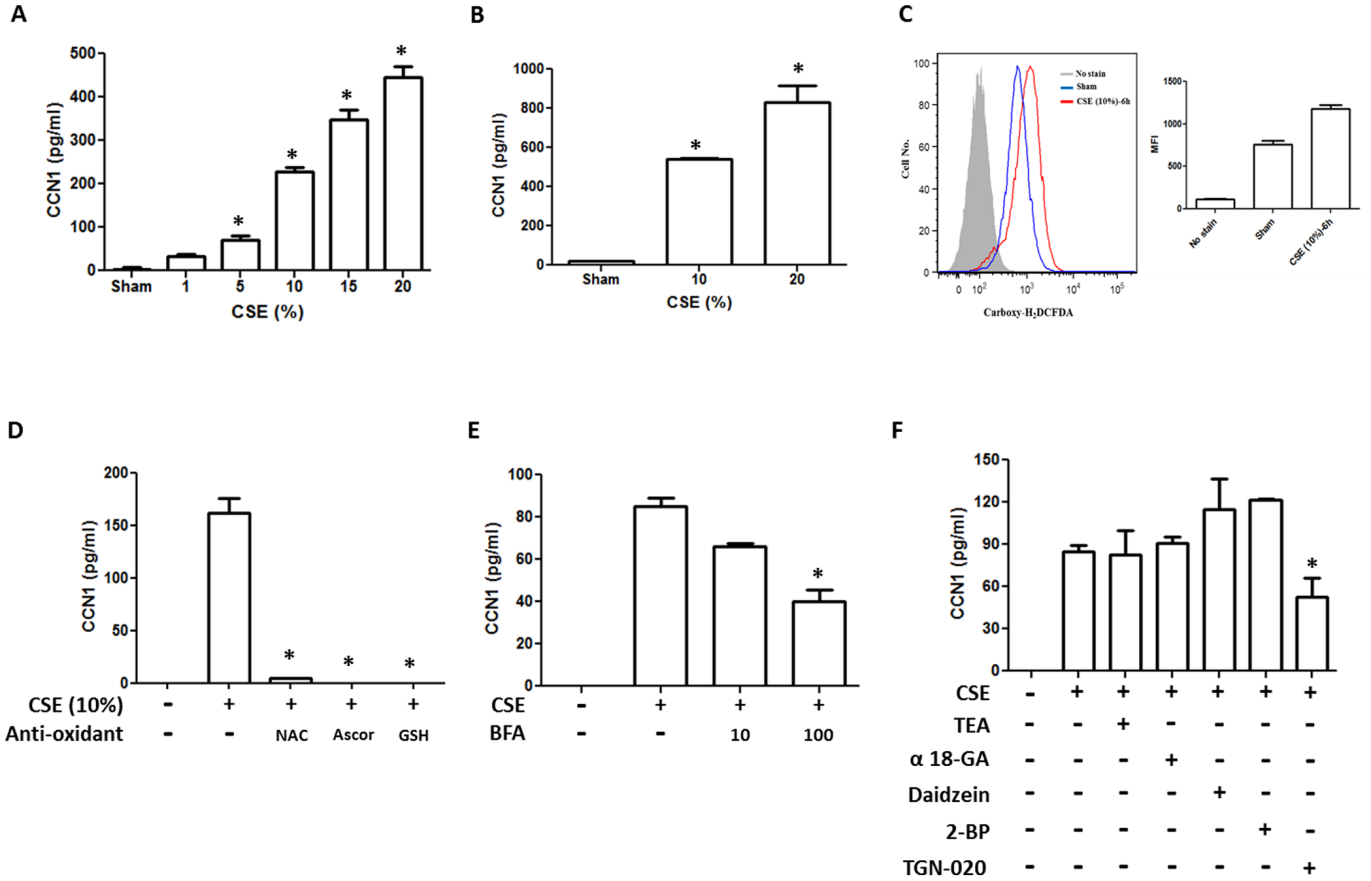
CSE induced CCN1 secretion in lung epithelial cells. (A) CCN1 secretion induced by CSE (1∼20%). Beas2B cells were treated with CSE in a dose-dependent manner. After 24 h, CCN1 level in the cell supernatant was detected using ELISA. (B) Mouse lung primary type II epithelial cells were exposed to CSE (10 or 20%). After 24 h, CCN1 in cell supernatant was detected as above. (C) Beas2B cells were treated with 10% CSE. After 6 h, ROS was detected using the carboxyl-H2DCFDA methods. (D) CSE induced CCN1 secretion was blocked by anti-oxidant reagents. Beas2B cells were pre-treated with three different anti-oxidant reagents (NAC, ascorbic acid and GSH, 1 mM respectively), followed by 10% CSE. After 24 h, cell supernatant was collected and CCN1 was detected as above. (E) CSE induced CCN1 release was inhibited by ER-Golgi secretion inhibitor, Brefeldin A (BFA), in a dose-dependent manner. Beas2B cells were treated with BFA, followed by CSE. After 24 h, CCN1 secretion was detected using ELISA. (F) Suppression of CSE induced CCN1 secretion by membrane channel protein inhibitors. Beas2B cells were treated with tetraethylammonium chloride (TEA, 100 µM) for aquaporin 1, α18-glycyrrhetinic acid (GA, 100 µM) for connexin, daidzein (50 µM) for caveolin, 2-bromopalmitate (2-BP, 100 µM) for palmitoylation, TGN-020(100 µM) for aquaporin 4. Then these cells were exposed to CSE. After 24 h, CCN1 secretion was determined. For all the above, supernatant from the same amount of cells was collected and subjected to ELSIA. *p<.05 All figures above represented three independent experiments with similar results.

CCN1 is a secreted protein and functions in an autocrine or paracrine manner [20]–[22]. Therefore, we next determined the level of secreted CCN1 in epithelial cells. We first characterized the CCN1 secretion after CSE treatment using Beas2B human lung epithelial cells. CSE induced CCN1 secretion from Beas2B cells in dose-dependent manner (Fig. 2A). We confirmed this observation using the primary lung epithelial cells isolated from mice. Consistently, 10% or 20% CSE robustly induced CCN1 secretion from primary lung epithelial cells (Fig. 2B). To determine whether CSE induced CCN1 via triggering oxidative stress, we first analyzed and confirmed the ROS generation after 10% CSE in Beas2B cells, using the carboxcy-H_2_DCFDA methods (Fig. 2C). Next, we pre-treated Beas2B cells with three different anti-oxidant reagents (NAC, ascorbic acid and GSH). The CSE induced secretion of CCN1 in these pre-treated cells was then re-analyzed. As shown in figure 2D, pretreatment of anti-oxidants abolished CSE induced CCN1 secretion. To determine the potential pathway of CCN1 secretion after CSE, we pre-treated the Beas2B cells with ER-Golgi inhibitor Brefeldin A (BFA) and a variety of membrane channel protein inhibitors. Lung epithelial cells communicate with the adjacent cells or itself via paracrine and/or autocrine manners. Channel proteins and junction proteins participate this communication [23]. To investigate the effect of channel/junction proteins in CCN1 secretion after CSE, we used a variety of channel/junction protein inhibitors to determine the major channel/junction protein which plays a role in CCN1 secretion. Therefore, we chose TEA as an aquaporin 1 inhibitor, α18-GA as a connexin (gap junction) inhibitor, daidzein and 2-BP as a caveolin inhibitor and TGF-020 as an aquaporin 4 inhibitor. Pre-treatment of BFA or TGN-020 (aquaporin 4 inhibitor) partially but significantly decreased the CSE induced CCN1 secretion, as shown in figure 2E and 2F respectively. This result suggested a potential secretive pathway involving ER-Golgi and membrane aquaporin 4.

### CSE Associated ER Stress Induced CCN1 Secretion in Lung Epithelial Cells

**Figure 3:**
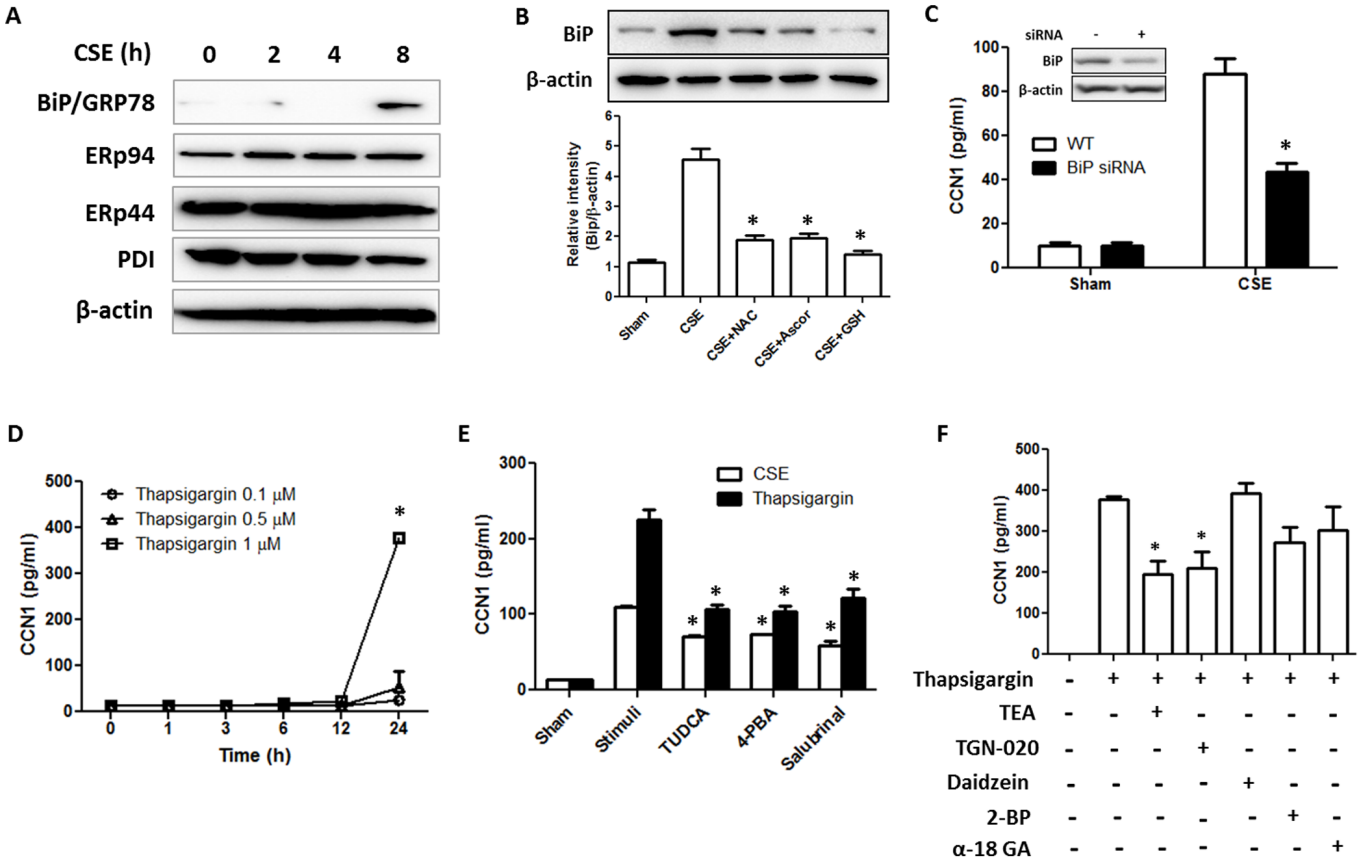
CSE augmented CCN1 secretion via induction of ER stress. (A) CSE up-regulated ER stress markers in a time-dependent manner in Beas2B cells. 10% CSE was used to treat Beas2B cells. After 24 h, cell lysate was subjected to Western Blot analysis. (B) CSE-induced BiP/GRP78 expression was suppressed by pre-treatment of 1 mM anti-oxidant, NAC, ascorbic acid and GSH, respectively. (C) Deletion of ER stress marker protein BiP suppressed CSE induced CCN1 secretion. Beas2B cells were transfected with BiP siRNA or control siRNA (WT). Cells were then exposed to CSE (10%). CCN1 secretion was determined as above. (D) Induction of CCN1 secretion by thapsigargin, an ER stress inducer, in a time and dose-dependent manner. Beas2B cells were treated with thapsigargin. CCN1 secretion was determined as above. (E) Inhibition of CSE or thapsigargin induced CCN1 secretion by ER stress inhibitors, tauroursodeoxycholic acid (TUDCA, 10 mM), 4-phenylbutyric acid (4-PBA, 5 mM) and salubrinal (100 µM). Beas2B cells were treated with these ER stress inhibitors followed by 10% CSE. CCN1 secretion was measured as above. (E) Suppression of CCN1 secretion by membrane channel protein inhibitors. Beas2B cells were treated with each inhibitor, followed by ER stress inducer thapsigargin (1 µM). CCN1 secretion was measured as above. *p<.05 All figures above represented two or three independent experiments with similar results.

CSE has been well documented to induce ER stress in a variety of diseases [24]–[26]. As shown above, CSE induced CCN1 secretion by epithelial cells involved in the ER-Golgi pathway (Fig. 2D). In our study, we first determined whether CSE induced ER stress in lung epithelial cells. After 10% CSE, as soon as 8 h, BiP/GRP78 was highly induced in Beas2B cells (Fig. 3A). Further, this CSE induced ER stress was associated with ROS, supported by the fact that pretreatment of anti-oxidant reagents suppressed the BiP up-regulation after CSE (Fig. 3B). Blocking BiP expression by transfection of BiP siRNA significantly suppressed CSE induced CCN1 secretion (Fig. 3C). To further confirm this observation, we induced ER stress using chemical inducer thapsigargin. Thapsigargin not only induced ER stress but also induced CCN1 secretion in a dose-dependent manner (Fig. 3D). Consistently, pretreatment of ER stress inhibitors, tauroursodeoxycholic acid (TUDCA), 4-phenylbutyric acid (4-PBA) and salubrinal, inhibited CCN1 secretion after CSE (Fig. 3E). To further confirm our observation in figure 2 that CCN1 secretion was via membrane channel aquaporin 4, we pretreated the cells with membrane channel inhibitors and followed by inducing CCN1 secretion using ER-stress inducer thapsigargin. CCN1 secretion induced by thapsigargin was also blocked by TGN-020, aquaporin 4 inhibitor, suggesting that aquaporin 4 may be a common pathway for CCN1 secretion induced by a variety of stimuli (Fig. 3F).

### IRE1-MAPK-JNK Pathway Regulated the CSE Induced CCN1 Secretion

**Figure 4:**
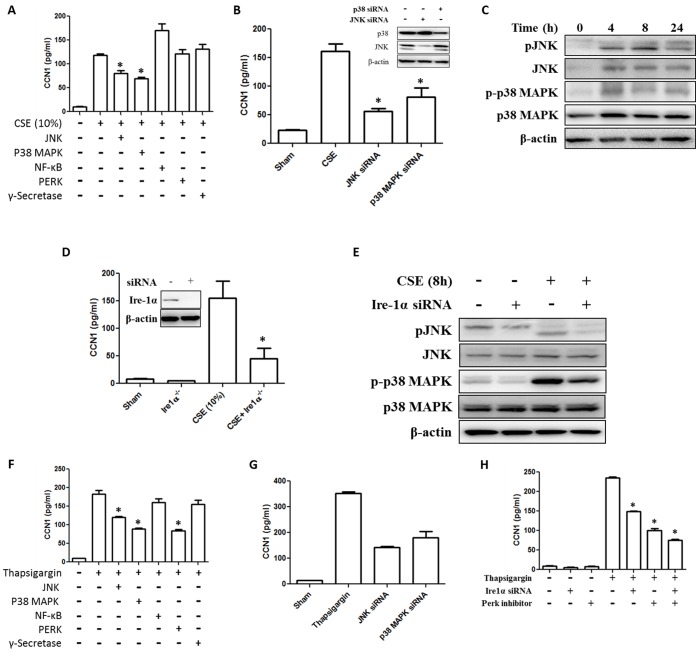
ER stress induced CCN1 secretion via Ire-1, MAPK, JNK pathways in lung epithelial cells. (A) CSE (10%)-induced CCN1 secretion in the presence of the inhibitors for ER stress related signaling. Beas2B cells were treated with JNK inhibitor II (10 µM), SB203580 (10 µM) for P38 MAPK, Bay11-7082 (10 µM) for NF-κB, GSK PERK (1 µM) for PERK and γ-secretase inhibitor (10 µM). Next, cells were exposed to CSE (10%) and CCN1 secretion was determined using ELISA as the above. (B) Reduced CCN1 secretion after CSE in the p38 MAPK and JNK siRNA transfected Beas2B cells. Beas2B cells were transfected with siRNA first. After 24 h, these cells were exposed to CSE (10%). After another 24 h, CCN1 secretion was determined using ELISA. (C) CSE (10%) induced JNK and p38 MAPK phosphorylation in Beas2B cells, determined by Western Blot analysis. (D) Deletion of Ire-1α by siRNA reduced CSE triggered CCN1 secretion. Beas2B cells were transfected with Ire-1α siRNA, followed by CSE (10%) exposure. CCN1 secretion was determined using ELISA as the above. Inset: the efficiency of Ire-1α deletion using siRNA. (E) Beas2B cells were transfected with Ire-1α siRNA or control siRNA, followed by CSE (10%). After 8 h, cell lysate was subjected to Western Blot analysis. pJNK, total JNK, p-p38 MAPK and total p38 MAPK were analyzed. (F) Beas2B cells were treated with ER stress inducer, thapsigargin (1 µM). CCN1 release was analyzed using ELISA. To determine the signaling pathways involved in the ER stress inducer triggered CCN1 release, we pre-treated the cells using a variety of pathway inhibitors as shown, followed by thapsigargin (1 µM). (G) Similarly, the above samples from (B) were subjected to ELISA to determine CCN1 secretion in p38 MAPK and JNK siRNA treated Beas2B cells. (H) Blocking of Ire-1α and/or PERK suppressed CCN1 secretion induced by thapsigargin. Beas2B cells were transfected with control siRNA or Ire-1α siRNA, treated with solvent or PERK inhibitor (10 µM), followed by thapsigargin (1 µM). CCN1 secretion was measured as above mentioned. *p<.05 All figures above represented two or three independent experiments with similar results.

ER stress has been shown to activate p38 MAPK and JNK [27]. To further investigate the CSE-induced ER stress involved in CCN1 secretion, we pre-treated Beas2B cells with a variety of inhibitors including inhibitors for JNK, p38 MAPK, NF-kappaB, PERK and γ-Secretase. After 10% CSE, inhibition of JNK and p38 MAPK resulted in less CCN1 secretion (Fig. 4A). In addition, we treated Beas2B cells with p38 MAPK or JNK siRNA, followed by 10% CSE. Consistently, Beas2B cells treated with p38 MAPK or JNK siRNA had reduced CCN1 secretion after CSE (Fig. 4B). Furthermore, CSE induced JNK and p38 MAPK phosphorylation in Beas2B cells (Fig. 4C). Previous report has shown that activated IRE1α leads to the phosphorylation of JNK and MAPK pathway [27]. In our studies, we first evaluated whether IRE1α involved in CSE induced CCN1 secretion. Deletion of IRE1α using IRE1α siRNA suppressed CSE induced CCN1 secretion markedly (Fig. 4D). Further, we confirmed that deletion of IRE1α prohibited the CSE induced phosphorylation of JNK and p38 MAPK (Fig. 4E). To determine whether JNK and p38 MAPK pathways are involved in the ER stress induced CCN1 secretion, we pre-treated the Beas2B cells with inhibitors to these pathways followed by ER stress inducer thapsigargin. Cells pretreated with inhibitors of p38 MAPK or JNK had less CCN1 secretion after ER stress inducer (Fig. 4F). Similar results were observed when using siRNA to inhibit p38 MAPK or JNK (Fig. 4G). Additionally, deletion of IRE1α by IRE1α siRNA also suppressed the ER stress induced CCN1 secretion (Fig. 4H).

### CSE-induced CCN1 Promoted IL-8 Secretion in Lung Epithelial Cells

**Figure 5:**
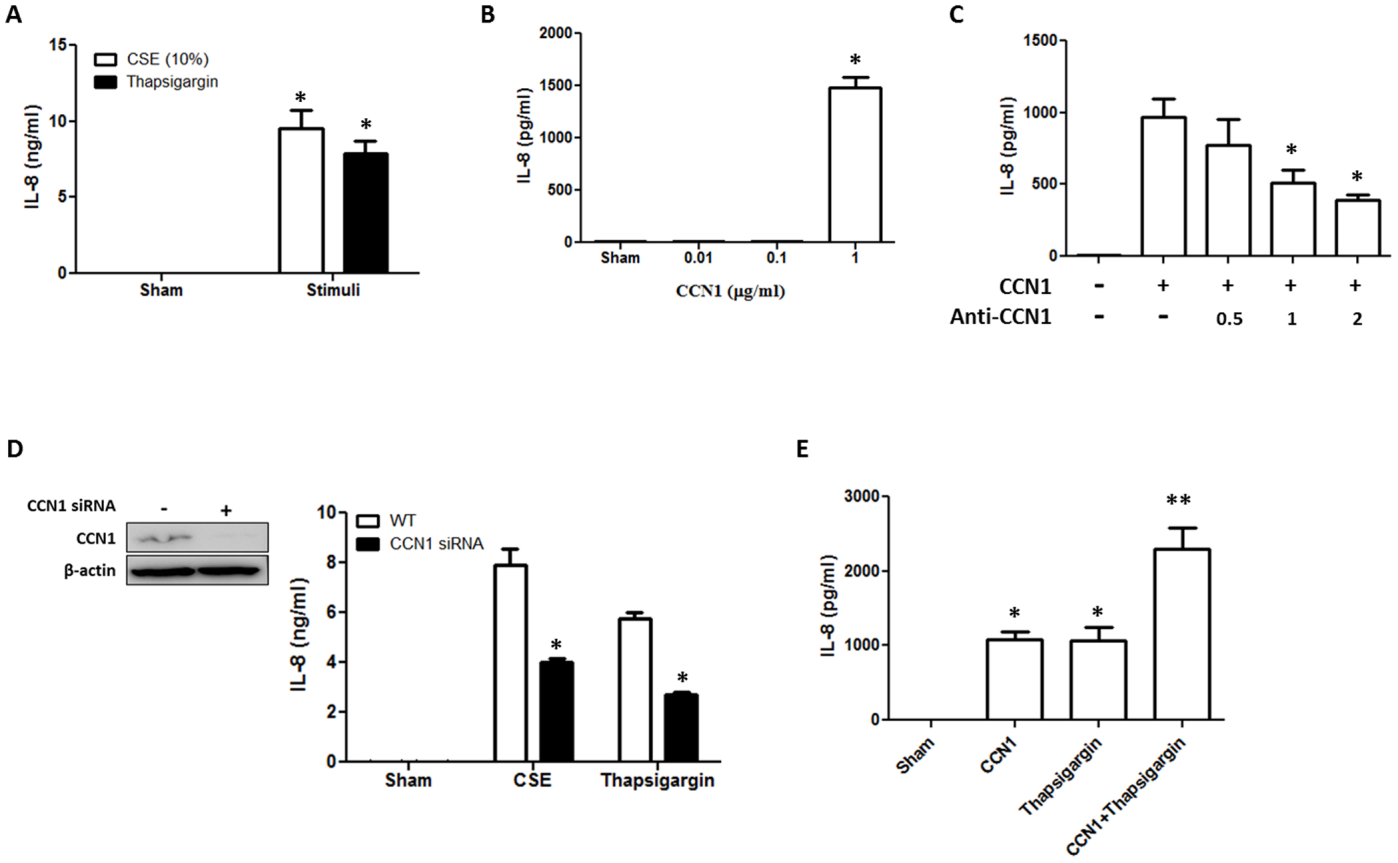
CSE-induced CCN1 release promoted IL-8 secretion from lung epithelial cells. (A) CSE (10%) and ER stress inducer, thapsigargin (1 µM), triggered IL-8 secretion from Beas2B cells. IL-8 secretion was analyzed using ELISA. (B) Recombinant CCN1-induced IL-8 secretion after 4 h exposure. (C) Neutralizing antibody anti-CCN1 (µg/ml) reduced CCN1-induced IL-8 secretion in a dose-dependent manner. (D) Deletion of CCN1 suppressed CSE or ER stress inducer thapsigargin induced IL-8 secretion. Beas2B cells were transfected with control siRNA or CCN1siRNA, followed by CSE (10%) or thapsigargin (1 µM). IL-8 secretion was analyzed using ELISA as above. Inset: the efficiency of CCN1 deletion using siRNA. (E) CCN1 and thapsigargin (1 µM) (4 h) synergistically enhanced IL-8 secretion in Beas2B cells. *p<.05, ** p<.05 All figures above represented two or three independent experiments with similar results.

CSE highly induced the expression and secretion of CCN1 in lung epithelial cells, therefore, we next investigated the function of secreted CCN1 in lung epithelial cells after CS. Lung inflammation has been well documented after CSE exposure [3] and ER stress [28]. IL-8 secretion is an important marker in early inflammation and is crucial for neutrophil recruitments [8]. Initially, we treated epithelial Beas2B cells with CSE and ER stress inducer thapsigargin. IL-8 secretion was robustly induced by both CSE and thapsigargin (Fig. 5A). Interestingly, 1 µg/ml recombinant CCN1 (with partial biological activity per manufacture) also induced IL-8 secretion (Fig. 5B). We further confirmed the CCN1 induced IL-8 secretion by blocking CCN1 using neutralizing antibody (Fig. 5C). Next, using CCN1 siRNA, we deleted the endogenous CCN1 in Beas2B epithelial cells (Fig. 5D inset). Deletion of CCN1 suppressed IL-8 secretion after CSE and ER stress inducer thapsigargin (Fig. 5D). Moreover, CCN1 and ER stress inducer thapsigargin synergistically stimulated IL-8 secretion (Fig. 5E). However, CCN1 and CSE failed to stimulate IL-8 secretion synergistically (Fig. S4).

### CCN1 Enhanced Il-8 Secretion via LRP6-Wnt Pathway

**Figure 6:**
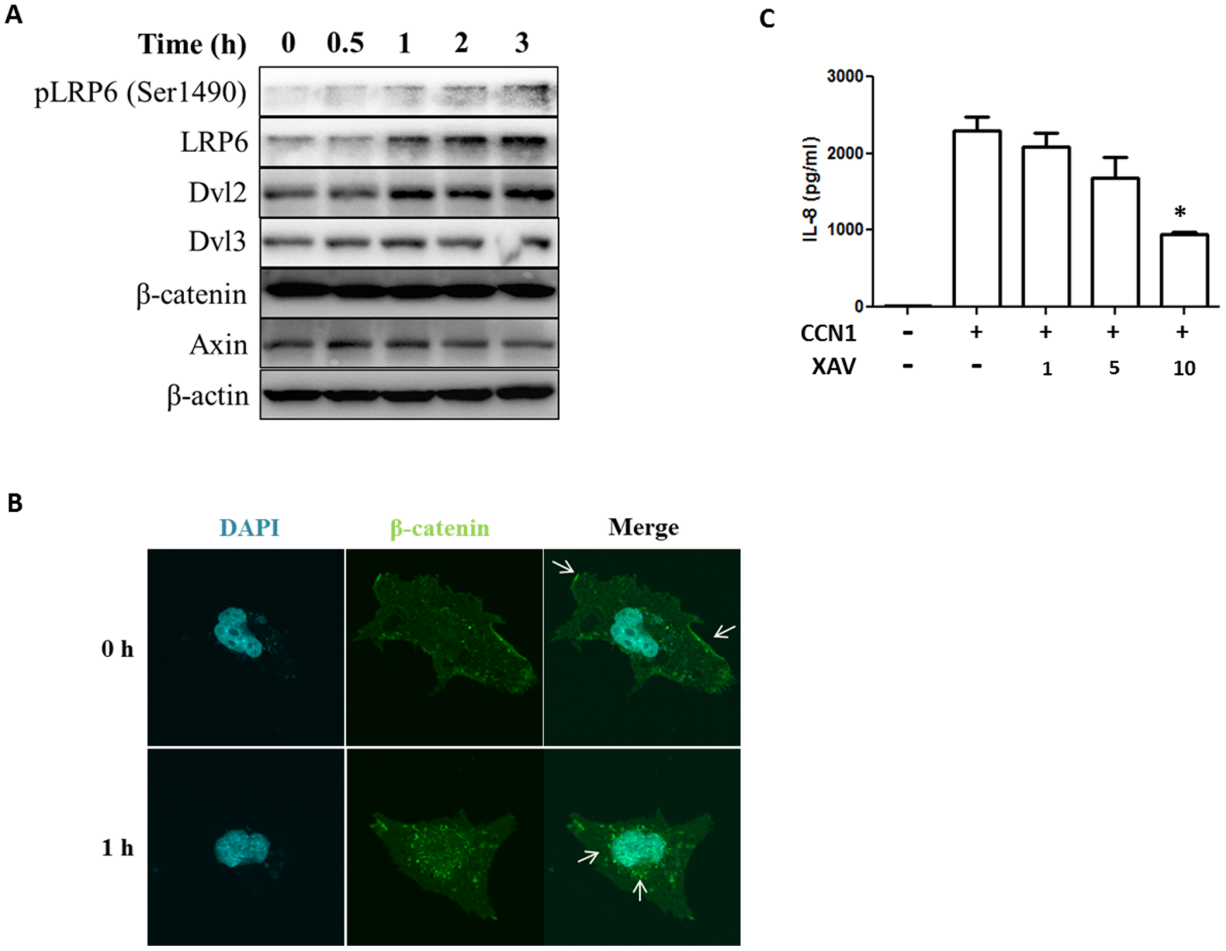
CSE enhanced IL-8 secretion via LRP6-Wnt pathway. (A) Expression of Wnt signaling associated proteins after CCN1 (1 µg/ml). Beas2B cells were treated CCN1, after the indicated time, cell lysate was subjected to Western Blot analysis. (B) Translocation of β-catenin from membrane to cytosol/nucleus after CCN1 (1 µg/ml). White arrow: β-catenin. (C) Wnt signaling inhibitor, XAV, suppressed CCN1 induced IL-8 secretion. Beas2B cells were treated with XAV at indicated dose (µM), followed by CCN1 recombinant protein (1 µg/ml). After 4 h, IL-8 in supernatant was determined using ELISA. *p<.05 All figures above represented two or three independent experiments with similar results.

To investigate the signal pathways involved in CCN1 triggered IL-8 secretion, we treated Beas2B cells with CCN1 recombinant protein in a time-dependent manner. CCN1 significantly induced Wnt pathway protein Dvl2, receptor LRP6 expression and phosphorylation (Fig. 6A). After exposed to CCN1 protein, Wnt pathway component β-catenin translocated from membrane to cytosol, perinuclear region and nucleus (Fig. 6B), suggesting the activation of Wnt pathway by CCN1. Pre-treatment of Wnt pathway inhibitor XAV suppressed the CCN1 induced IL-8 secretion from Beas2B cells (Fig. 6C).

## Discussion

**Figure 7:**
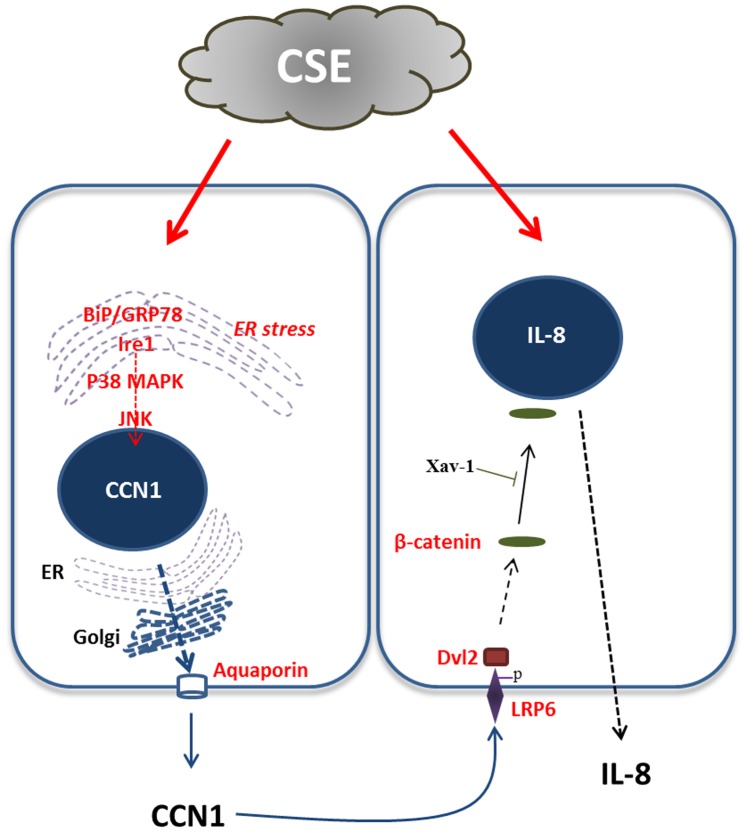
CSE-induced CCN1 augments IL-8 secretion. CCN1 was highly up-regulated by CSE, both expression and secretion in lung epithelial cells. This induction was largely ER stress dependent. Augmented matrix CCN1 next acted on the adjacent epithelial cells in an autocrine or paracrine manner. CCN1 first phosphorylated Wnt pathway receptor LRP6. Next the Wnt pathway components were increased, such as Dvl2. β-catenin translocated from cell surface to cytosol and nucleus, subsequently triggered the release of IL-8 from lung epithelial cells.

Inflammation has been recognized as a central stage for many CS associated diseases, characterized by increased neutrophils, lymphocytes, macrophages and pro-inflammatory cytokines/chemokines [29]. Among all the cytokines and chemokines, robustly increased IL-8 levels are found in sputum from patients with COPD [8], [30] and particularly at the time of exacerbation [31], [32]. In smokers, the level of IL-8 (both protein and mRNA), but not other chemokines, is markedly higher comparing with the control [33]–[34]. The up-regulation and secretion of IL-8 is regulated transcriptionally by several transcription factors through MAPK pathways [35]. However, the mediators between CSE and IL-8 release have not been completely explored. Our study identified a novel ECM molecule which regulates the CSE induced IL-8 secretion. We further illustrated a novel pathway connecting the initial noxious stimulation (CSE) to the end effectors (IL-8) in the pathogenesis of lung inflammation. As shown in figure 7, CCN1, an early stress-response gene product, was highly up-regulated by CSE, both expression and secretion in lung epithelial cells. This induction was largely ROS and ER stress dependent (Fig. 7). Augmented matrix CCN1 next acted on the adjacent epithelial cells in an autocrine or paracrine manner. CCN1 first phosphorylated Wnt pathway receptor LRP6. Next the Wnt pathway components were increased, such as Dvl2. β-catenin translocated from cell surface to cytosol and nucleus, subsequently triggered the release of IL-8. This study potentially suggested a crucial central molecule bridging the noxious stimuli to the pro-inflammatory chemokines. Furthermore, CCN1, as a secreted molecule, connected lung epithelial cells to the matrix. By modulating the release of IL-8, it further provided a cross-talk between epithelial cells and lung inflammatory cells. CCN1 is likely a central signal dispatcher controlling the direction of lung pathogenesis, such as inflammation, apoptosis and fibrosis. In fact, previous reports have shown important roles of CCN1 in lung epithelial cell apoptosis after oxidative stress [12]. The findings in our current report further extended the importance of this ECM molecule in triggering lung inflammation. Additionally, this study indicated an important function of lung epithelial cells on the development of lung inflammation. As a communicator between lung parenchymal cells and inflammatory cells, as well as a connector between intracellular components and ECM, CCN1 indicated a potential cellular target for therapy development in a variety of lung diseases associated with CS.

Our current studies focused on the cellular and molecular aspects of CCN1 associated IL-8 release. We showed that ROS and ER stress were highly involved in CSE triggered CCN1 up-regulation and secretion. It is very possible that other oxidative stress and ER stress inducers also stimulate CCN1 release, such as hyperoxia. In fact, this has been hypothesized both in vitro and in vivo using hyperoxia model [12], [13]. CS is a long term (chronic) process. Continuous and long-lasting stimulation with CS or CSE may have different effects on CCN1 secretion comparing to acute stimuli, such as hyperoxia. Our results on the signaling pathways involved CSE induced CCN1 secretion were based on in-vitro assays. Chronic CSE or ER stress inducer treatment caused cyto-toxicity in cultured cells, even at a relatively low dose (Fig. S2, S3). Thus, the chronic effects of CS on CCN1 release in vivo require future studies. Our results should not be extrapolated automatically to the animal models chronically exposed to CS, neither to chronic smokers.

There are also a number of limitations in this current study: First, the recombinant protein of CCN1 may only carry a partial bioactivity and thus, we used a relatively larger dose to treat Beas2B cells in vitro. In reality, the absolute amount of CCN1 protein secreted into ECM largely depends on the number of cells in the micro-environment. Second, although it induces ER stress, CSE may function via other alternative signaling pathways. Due to the complexity of the chemical components in CSE, we are not able to fully address its signaling pathways besides ER stress, in current study.

Future directions on the role of CCN1 in CS induced IL-8 release in vivo include the following: 1) Examining the level of CCN1 and IL-8 using BALF obtained from mice exposed to CS at different time points; 2) Comparing the time points of CCN1 peak level and IL-8 peak level; 3) Blocking CCN1 with neutralizing antibodies in vivo and examining the IL-8 secretion in BALF, in the presence and absence of CS exposure; 4) Administration of bioactive CCN1 in vivo and examining the IL-8 secretion in BALF, in the presence and absence of CS exposure; We hypothesize that CCN1 release in vivo is associated with acute exposure of CS (early stress) but will be blunted after chronic exposure. CCN1 up-regulation in lung tissue after three month exposure of CS was not as robust as those after the short time exposure (Fig. S1 and not shown). If this hypothesis is proven true, clinically, the CCN1 induction could be associated with only acute inflammation and exacerbation in COPD patients. Therefore, additionally, future studies also include: 1) to measure CCN1 level in plasma from mice which are exposed to CSE chronically and acutely; 2) to determine the probability of CCN1 as a biomarker for COPD exacerbation.

In summary, CCN1 expression and secretion from lung epithelial cells are markedly induced by CS and CSE. This elevated matrix CCN1 plays a crucial role in IL-8 release from epithelium after CSE exposure.

## Supporting Information

Figure S1 **CCN1 expression in homogenized lung tissue from mice exposed to room air or CS.** C57/B6J mice (6–8 weeks, male) were exposed to room air or CS as previously described [18]. After three months, lung tissue was obtained and homogenized. Lysate was subjected to western blot analysis.
(TIF)

Figure S2 **Dose-dependent effect of CSE on cell viability.** (A) Beas2B cells were exposed to CSE in different concentrations. Cell viability was determined after 3, 9 and 24 h. (B) Primary mouse type II lung epithelial cells were treated with 1, 5 and 10% of CSE, after 24 h, cell viability was determined.
(TIF)

Figure S3 **Dose-dependent effects of ER stress inducer, thapsigargin, on cell viability.** Beas2B cells were treated with 0.1, 0.5 and 1 (µM) of thapsigargin. After 24 h, cell viability was determined.
(TIF)

Figure S4 **CCN1 and CSE have no synergistic effect on IL-8 secretion.** Beas2B cells were treated with CCN1 (1 µg/ml), CSE (10%) or the combination of these two. IL-8 secretion was determined using ELISA.
(TIF)
