# Supplementary figures and images for: CCN1 Secretion Induced by Cigarette Smoking Extracts Augments IL-8 Release from Bronchial Epithelial Cells
Source: PLoS One. 2013 Jul 9;8(7):e68199. doi: 10.1371/journal.pone.0068199 (PMC3706594; doi:10.1371/journal.pone.0068199)

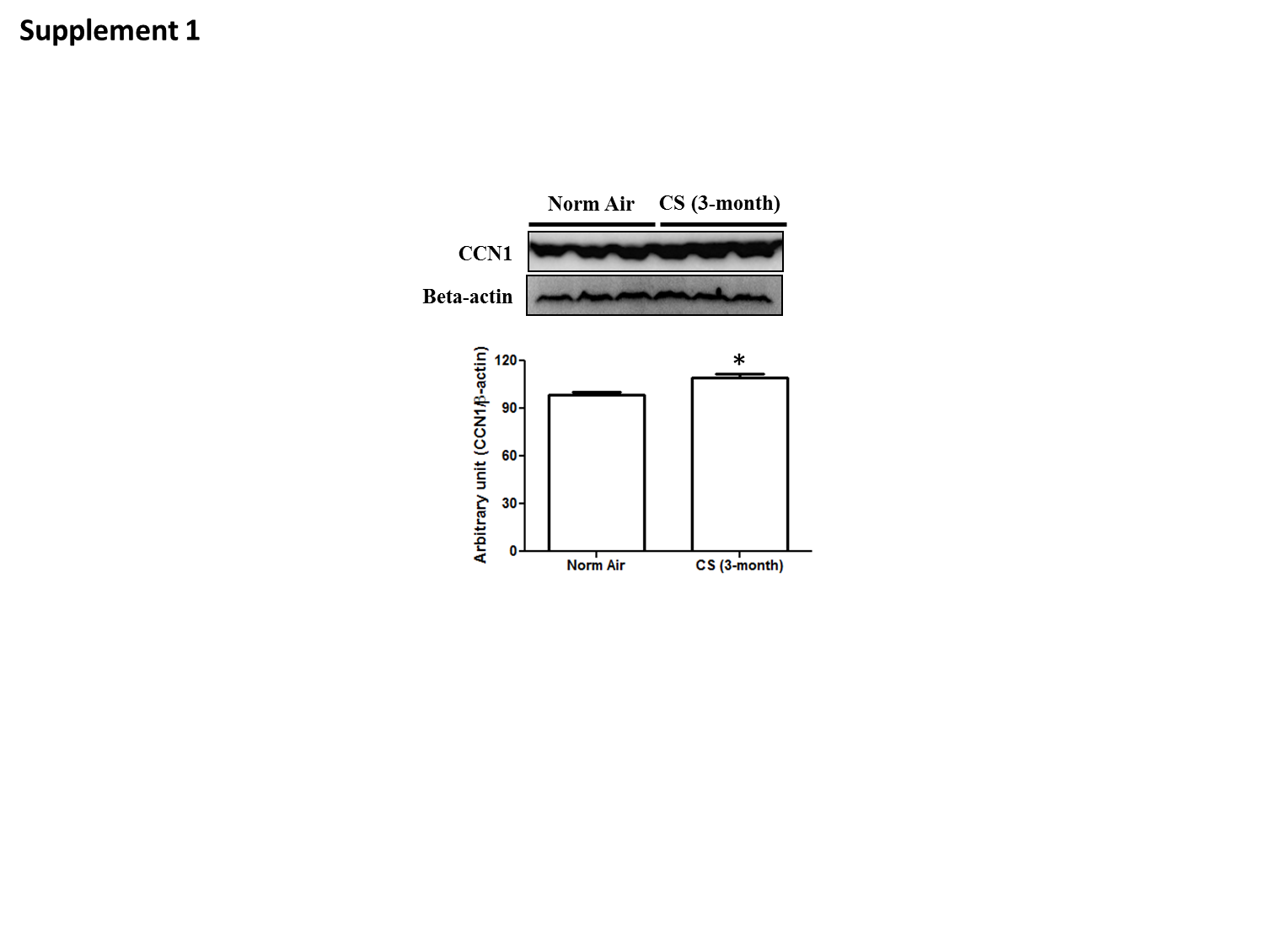

Supplement: Figure S1 — CCN1 expression in homogenized lung tissue from mice exposed to room air or CS. C57/B6J mice (6–8 weeks, male) were exposed to room air or CS as previously described [18]. After three months, lung tissue was obtained and homogenized. Lysate was subjected to western blot analysis. (TIF) [file pone.0068199.s001.tif]

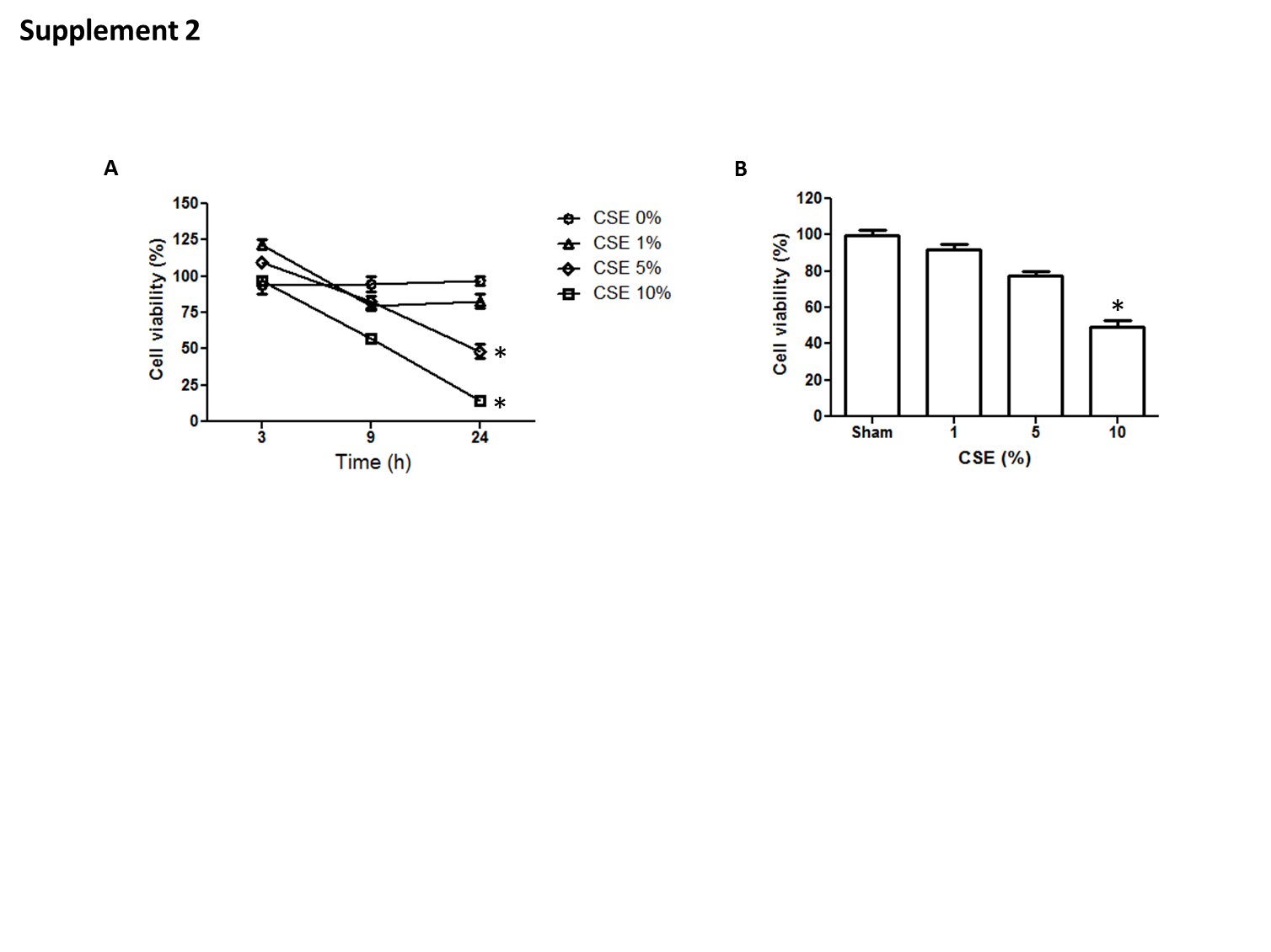

Supplement: Figure S2 — Dose-dependent effect of CSE on cell viability. (A) Beas2B cells were exposed to CSE in different concentrations. Cell viability was determined after 3, 9 and 24 h. (B) Primary mouse type II lung epithelial cells were treated with 1, 5 and 10% of CSE, after 24 h, cell viability was determined. (TIF) [file pone.0068199.s002.tif]

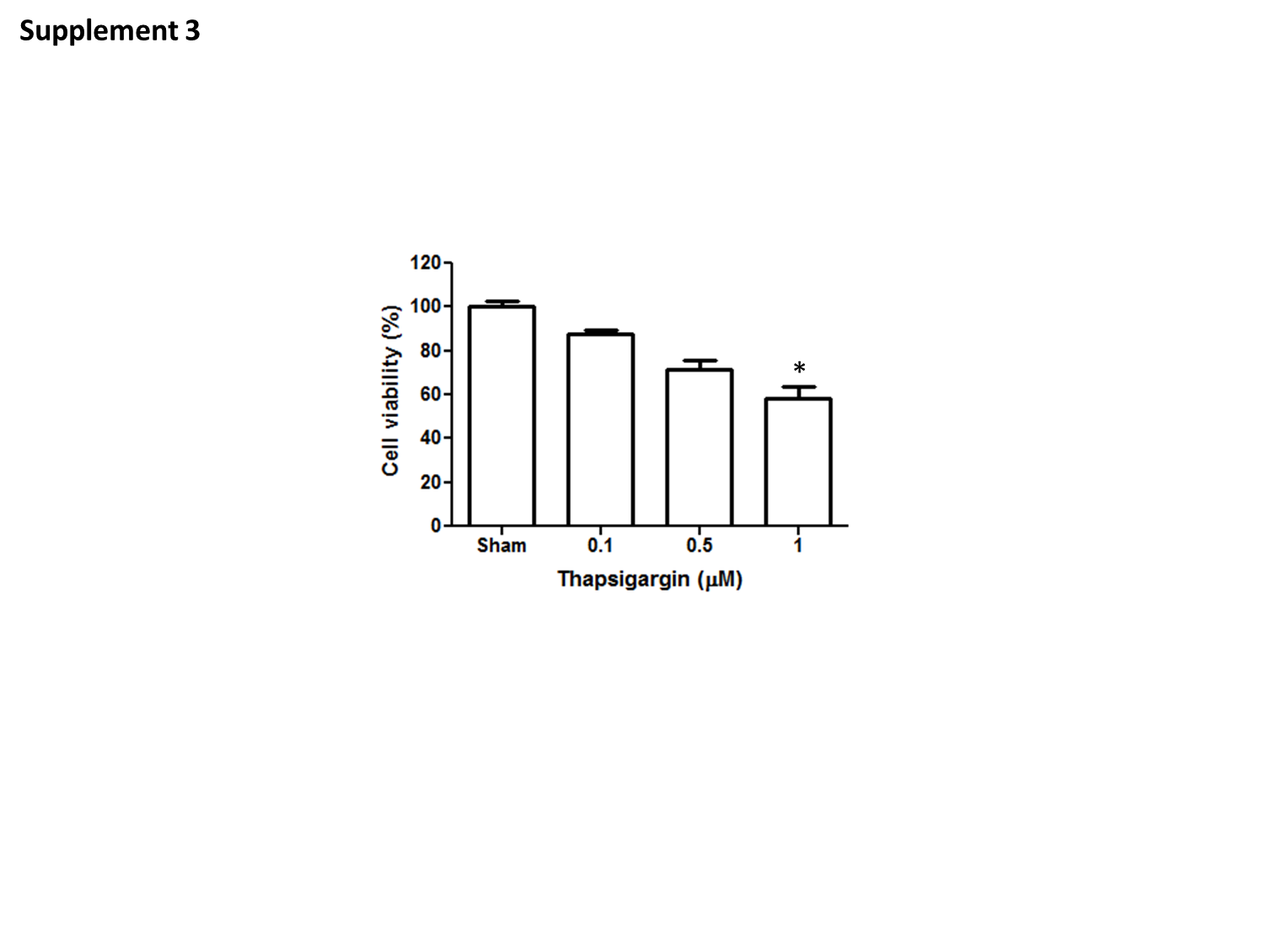

Supplement: Figure S3 — Dose-dependent effects of ER stress inducer, thapsigargin, on cell viability. Beas2B cells were treated with 0.1, 0.5 and 1 (µM) of thapsigargin. After 24 h, cell viability was determined. (TIF) [file pone.0068199.s003.tif]

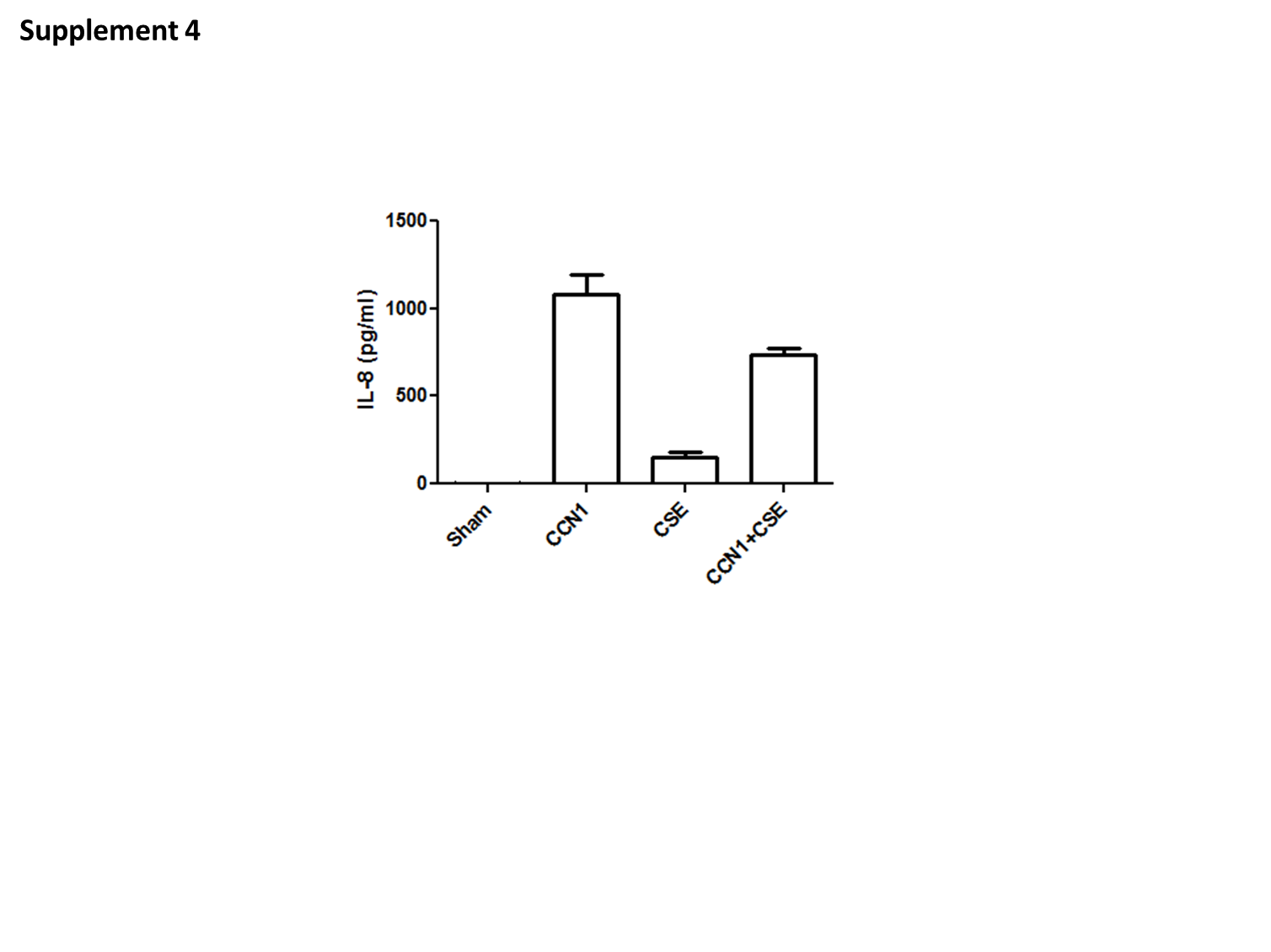

Supplement: Figure S4 — CCN1 and CSE have no synergistic effect on IL-8 secretion. Beas2B cells were treated with CCN1 (1 µg/ml), CSE (10%) or the combination of these two. IL-8 secretion was determined using ELISA. (TIF) [file pone.0068199.s004.tif]
